# Molecular characterization of emerging recombinant African swine fever virus of genotype I and II in Vietnam, 2023

**DOI:** 10.1080/22221751.2024.2404156

**Published:** 2024-09-11

**Authors:** Kyungmoon Lee, Thi Thu Hang Vu, Minjoo Yeom, Viet Dung Nguyen, Thi Tam Than, Van Tam Nguyen, Dae Gwin Jeong, Aruna Ambagala, Van Phan Le, Daesub Song

**Affiliations:** a Department of Virology, College of Veterinary Medicine and Research Institute for Veterinary Science, Seoul National University, Seoul, Republic of Korea; b Institute of Veterinary Science and Technology, Vietnam Union of Science and Technology Association, Hanoi, Vietnam; c College of Veterinary Medicine, Vietnam National University of Agriculture, Hanoi, Vietnam; d Korea Research Institute of Bioscience and Biotechnology, Daejeon, Republic of Korea; e Canadian Food Inspection Agency, National Centre for Foreign Animal Disease, Winnipeg, Canada

**Keywords:** African swine fever virus, recombinant strains, genetic markers, diagnostic markers, genotype I and II, homologous recombination

## Abstract

African swine fever virus (ASFV) recombinant strains pose new challenges for diagnosis and control. This study characterizes genotype I and II recombinant ASFV strains identified in northern Vietnam in 2023 through whole-genome sequencing and comparative genomic analysis. Seven ASFV-positive samples from six provinces were analyzed, with recombinant strains detected in Bac Giang, Phu Tho, and Vinh Phuc provinces. Isolates showed hemadsorption positivity despite having genotype I B646L, indicating their recombinant nature. Genome-wide analysis revealed 19 recombination breakpoints consistent with Chinese recombinant strains. Vietnamese isolates shared 99.86-99.98% nucleotide identity with Chinese recombinants, forming a distinct monophyletic group. Comparative analysis identified 50 SNPs and INDELs, with 39 variations found across Vietnamese strains, distinguishing them from Chinese isolates. Unique genetic markers in C962R, I329L, and MGF 505-11L genes distinguished Vietnamese recombinants from Chinese counterparts, while mutations in C122R and NP1450L differentiated all recombinants from parental genotypes. The central variable region (CVR) of the B602L gene showed diversity among Vietnamese isolates, while the I73R-I329L intergenic regions were recognized as in the IGR2 group. This study enhances understanding of recombinant ASFV evolution through homologous recombination and identifies new genetic markers for improved detection and characterization. The observed genetic diversity highlights challenges for existing diagnostic methods and vaccine development, emphasizing the need for continued surveillance and research into the functional implications of these genetic variations on ASFV pathogenicity and transmissibility.

African swine fever (ASF) is a viral haemorrhagic disease caused by the African swine fever virus (ASFV), which is highly contagious and fatal in domestic pigs and wild boars [1]. ASFV is a large double-stranded DNA virus belonging to the family *Asfarviridae*, and 24 genotypes have been identified based on the B646L gene [1]. Only genotypes I (G1) and II (G2) are widespread outside Africa, and G2 has rapidly spread to Europe and Asia since the Georgian outbreak in 2007 [2–4]. In 2018, the G2 ASFV was first detected in China and spread to 15 other Asian countries [3, 4]. The identification of G1 ASFV was reported in China in 2021, resulting in a co-circulating environment of G1 and G2 ASFV [5]. The coexistence of both G1 and G2 ASFV led to the emergence of recombinant strains combining elements from both genotypes. These recombinants were first observed in China and subsequently in Vietnam in 2023 [6, 7]. In contrast to China, Vietnam had only reported G2 ASFV cases, with no detection of G1 ASFV, even with the emergence of recombinant strains [7].

Recombinant ASFV poses new challenges for diagnosis and epidemiological tracking. Traditional molecular markers, including B646L, B602L, EP402R, and the I73R-I329L intergenic region (IGR), are less effective in accurately characterizing these novel recombinants due to the mixing of genes from different genotypes [1, 4, 8]. Consequently, new molecular markers specific to recombinant ASFV strains are urgently needed.

In this study, we conducted a comprehensive molecular characterization of recombinant ASFV strains identified in northern Vietnam in 2023. Through whole-genome sequencing and comparative genomic analysis, we elucidated their genetic composition and evolutionary relationships with previously identified ASFV strains. Our in-depth molecular analysis discovered unique genetic markers that not only distinguish Vietnamese recombinant strains from Chinese recombinants but also differentiate these recombinant strains from parental G1 and G2 viruses.

Whole blood samples were collected from unvaccinated pigs showing acute ASF symptoms including fever and abortion in six northern Vietnam provinces (Yen Bai, Bac Giang, Thai Nguyen, Phu Tho, Vinh Phuc, and Hai Duong) in September 2023. All seven samples were submitted to our laboratory for routine ASFV diagnostic testing and detected as positive using real-time PCR (Table S1). Genotyping and serogrouping revealed that exclusively samples from Bac Giang, Phu Tho, and Vinh Phuc provinces showed a unique combination: G1 for B646L, G2 for E183L and CP204L, and serogroup VIII for EP402R (Figure S1). This indicated the presence of recombinant ASFV strains in northern Vietnam.

To confirm these findings, we isolated the viruses using porcine alveolar macrophages. The isolates showed hemadsorption (HAD) positive reactions (Figure S2), despite having G1 based on the B646L gene, typically associated with HAD negativity. This HAD positivity, characteristic of G2 strains, further supports the recombinant nature of these ASFV isolates, combining features of G1 and G2. Subsequently, we performed whole-genome sequencing on these isolates. The complete genome sequences were deposited in GenBank: VNUA/rASFV/BG2/2023 (BG2/23) from Bac Giang (PP810979), VNUA/rASFV/PT2/2023 (PT2/23) from Phu Tho (PP810980), and VNUA/rASFV/VP1/2023 (VP1/23) from Vinh Phuc (PP810981). Comparative sequence and phylogenetic analysis showed that the Vietnamese recombinant ASFV strains shared a high nucleotide identity of 99.86–99.98% with Chinese recombinant strains from 2021–2022, forming a distinct monophyletic group between genotype I and II ASFVs (Table S2, Figure S3).

To further characterize these recombinant strains, we performed genome-wide recombination analysis using RDP4 software and comparative sequence analysis. Our analysis identified 19 recombination breakpoints distributed across the genome (Figure S4, Table S3). Notably, these breakpoints were consistent with those previously reported in Chinese recombinant ASFV strains [6]. The recombinant strains exhibited a mosaic pattern of sequence similarity, characterized by alternating regions of high identity to G1 or G2 (Table S4). However, the high genetic stability of ASFV hinders the precise determination of the origin of G2 segments when comparing G2-derived regions with Vietnamese and Chinese G2 sequences (data not shown).

Comparative genomic analysis, using the IM/DQDM/2022 strain which showed the highest nucleotide identity with Vietnamese isolates, identified 50 single nucleotide polymorphisms (SNPs) and insertion/deletions (INDELs) among recombinant ASFV strains (Figure 1A). Of these, 11 differences were observed between Chinese isolates, while 39 distinguished Vietnamese from Chinese strains. Among the Vietnam-specific variations, 19 were in IGRs, 11 were non-synonymous substitutions, and 9 were synonymous substitutions in coding sequences (Table S5). Our analysis identified two distinct sets of genetic markers in recombinant ASFV strains. First, SNPs in C962R, I329L, and MGF 505-11L genes were unique to Vietnamese recombinant strains, distinguishing them from their Chinese counterparts. Second, mutations in C122R and NP1450L genes were consistently observed across all recombinant ASFV isolates, differentiating them from both G1 and G2 parental strains (Figure 1B). Among these various molecular markers, only those in I329L, NP1450L, and C122R resulted in amino acid changes, while the others were synonymous. All identified SNPs and INDELs, including these specific mutations, were subsequently confirmed by Sanger sequencing, reinforcing their reliability as diagnostic markers (Table S6).

**Figure 1.**
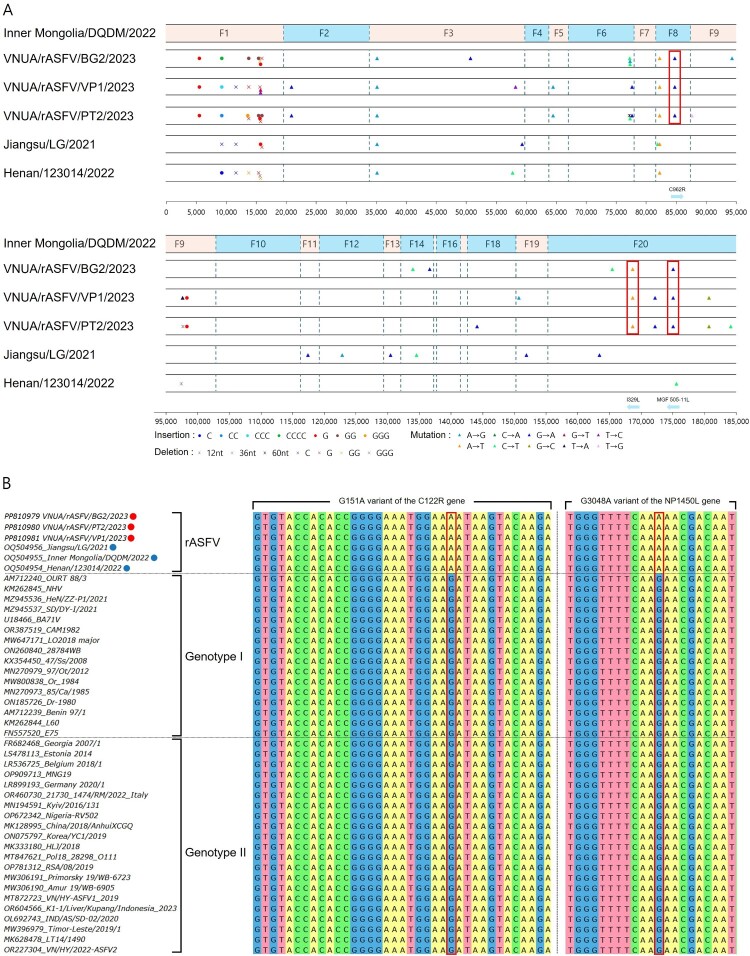
Genomic comparison of recombinant African Swine Fever Virus (ASFV) strains. (A) Schematic representation of genomic differences between Vietnamese and Chinese recombinant ASFV strains. The genome is divided into fragments (F1-F20) based on recombination breakpoints. Red fragments indicate regions derived from genotype I, while blue fragments are from genotype II. Red boxes highlight unique mutations in Vietnamese strains. (B) Multiple sequence alignment of C122R and NP1450L gene regions across various ASFV strains. Red dots represent Vietnamese recombinant ASFV strains, while blue dots indicate Chinese ones. The alignment shows the G151A variant in C122R and the G3048A variant in NP1450L, which are consistently observed in all recombinant ASFV isolates, distinguishing them from genotype I and II parental strains.

Analysis of the central variable region (CVR) of the B602L gene revealed diverse patterns among Vietnamese isolates (Figure S5). BG2/23 was identical to Chinese recombinant isolates, while VP1/23 showed a two-nucleotide change modifying one tetrameric repeat, and PT2/23 exhibited a 12-nucleotide deletion removing one tetrameric repeat. Comparison with previously reported Vietnamese recombinant ASFV strains showed that most had either identical sequences or variations in 1–2 tetrameric repeats, with one strain displaying eight additional tetrameric repeats. Examination of the I73R-I329L IGR demonstrated that these new recombinant isolates belong to the IGR2 group, consistent with most previously reported G2 ASFV strains from China and Vietnam (Figure S6).

Our study provides insights into the genetic characteristics of recombinant ASFV strains in Vietnam, revealing their close relationship with Chinese recombinant strains while identifying unique genetic markers. Consistent with previous reports of high genetic relatedness between Chinese and Vietnamese ASFV genomes, the observed high nucleotide identity and shared recombination patterns further underscore the risk of transboundary transmission and suggest a common evolutionary origin for these recombinant strains [6, 7, 9].

Genome-wide recombination analysis identified 19 breakpoints, consistent with those in Chinese recombinant ASFV strains [6]. These recombination events likely occur through strand switching during DNA replication in the cytoplasm, a process similar to poxvirus replication involving the formation of head-to-head concatemers [10–12]. This mechanism, facilitated by the high degree of sequence identity between ASFV strains, generates mosaic genomes, increasing the genetic diversity of ASFV.

The genetic markers identified in this study provide valuable insights into the evolution and spread of recombinant ASFV strains. The mutations in C122R and NP1450L, common to all recombinant strains, suggest a shared evolutionary path distinct from their parental genotypes. Meanwhile, the unique SNPs in C962R, I329L, and MGF 505-11L in Vietnamese strains indicate regional diversification. These strains were isolated from three geographically close provinces, suggesting a potential common origin due to unrestricted domestic pig trading between these areas.

The NP1450L gene encodes a viral RNA polymerase subunit, while the I329L gene is involved in inhibiting the NF-κB transcription factor and is associated with the expression of the MGF110 gene family [13,14]. While the function of C122R gene is not fully characterized, mutations consistently observed in these genes across multiple isolates may affect these functions, and further research is needed to understand their impact on the adaptive strategies of these recombinant ASFV strains and inform future control measures.

The diversity observed in the B602L gene, contrasted with the consistency in the I73R-I329L IGR, presents an interesting paradigm of genomic plasticity versus conservation in these recombinant ASFV strains. This pattern may reflect different evolutionary pressures on various parts of the viral genome, with some regions more susceptible to change than others [15].

In conclusion, our study enhances understanding of recombinant ASFV evolution through homologous recombination and provides new tools for their detection and characterization. The genetic diversity observed poses challenges for existing diagnostic methods and vaccine development, emphasizing the need for continued surveillance and research. Future studies should focus on the functional implications of these genetic variations and their potential impact on ASFV pathogenicity and transmissibility, along with comprehensive surveillance to assess the prevalence and spread of these recombinant strains in Vietnam and neighbouring countries.

## Supplementary Material

Figure S6.jpg

Supplementary materials_revision.docx

Figure S3.jpg

Figure S4.jpg

Figure S2.jpg

Figure S5.jpg

Figure S1.jpg
