# Supplementary figures and images for: Molecular characterization of emerging recombinant African swine fever virus of genotype I and II in Vietnam, 2023
Source: Emerg Microbes Infect. 2024 Sep 11;13(1):2404156. doi: 10.1080/22221751.2024.2404156 (PMC11421136; doi:10.1080/22221751.2024.2404156)

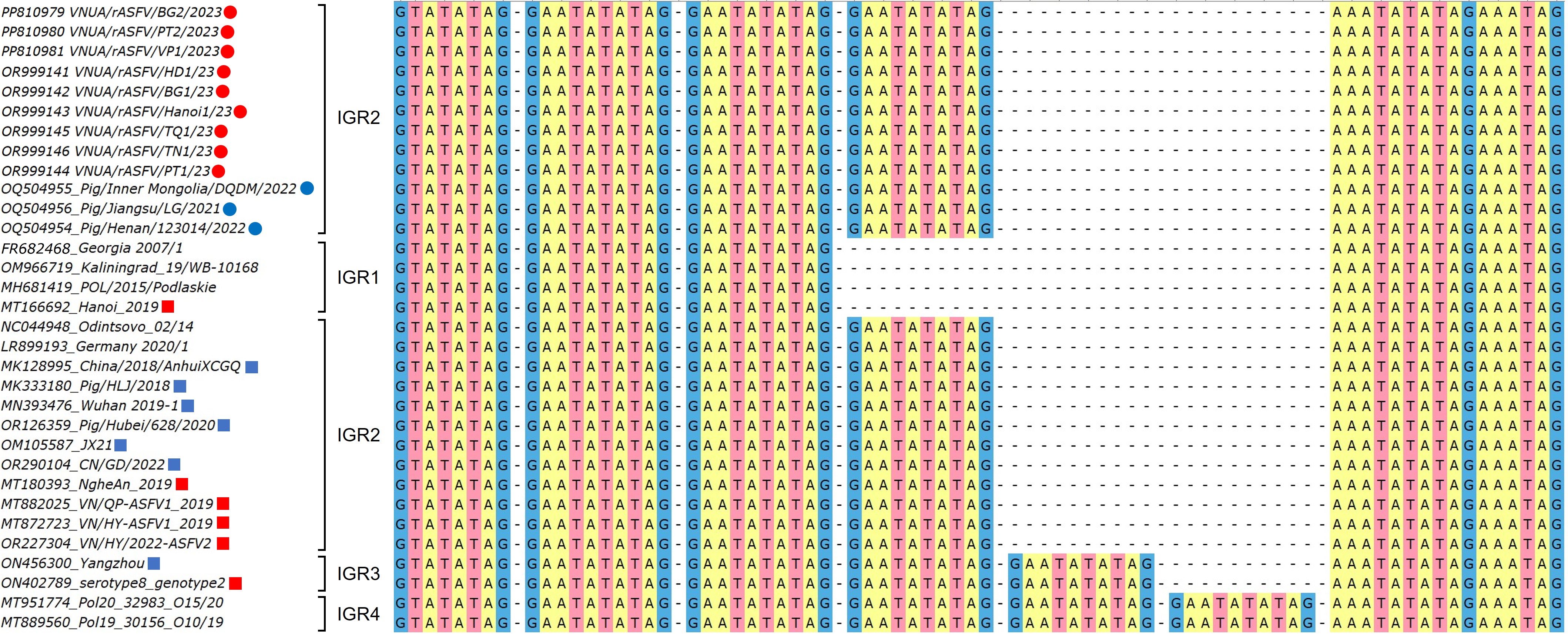

Supplement: Figure S6.jpg [file TEMI_A_2404156_SM7740.jpg]

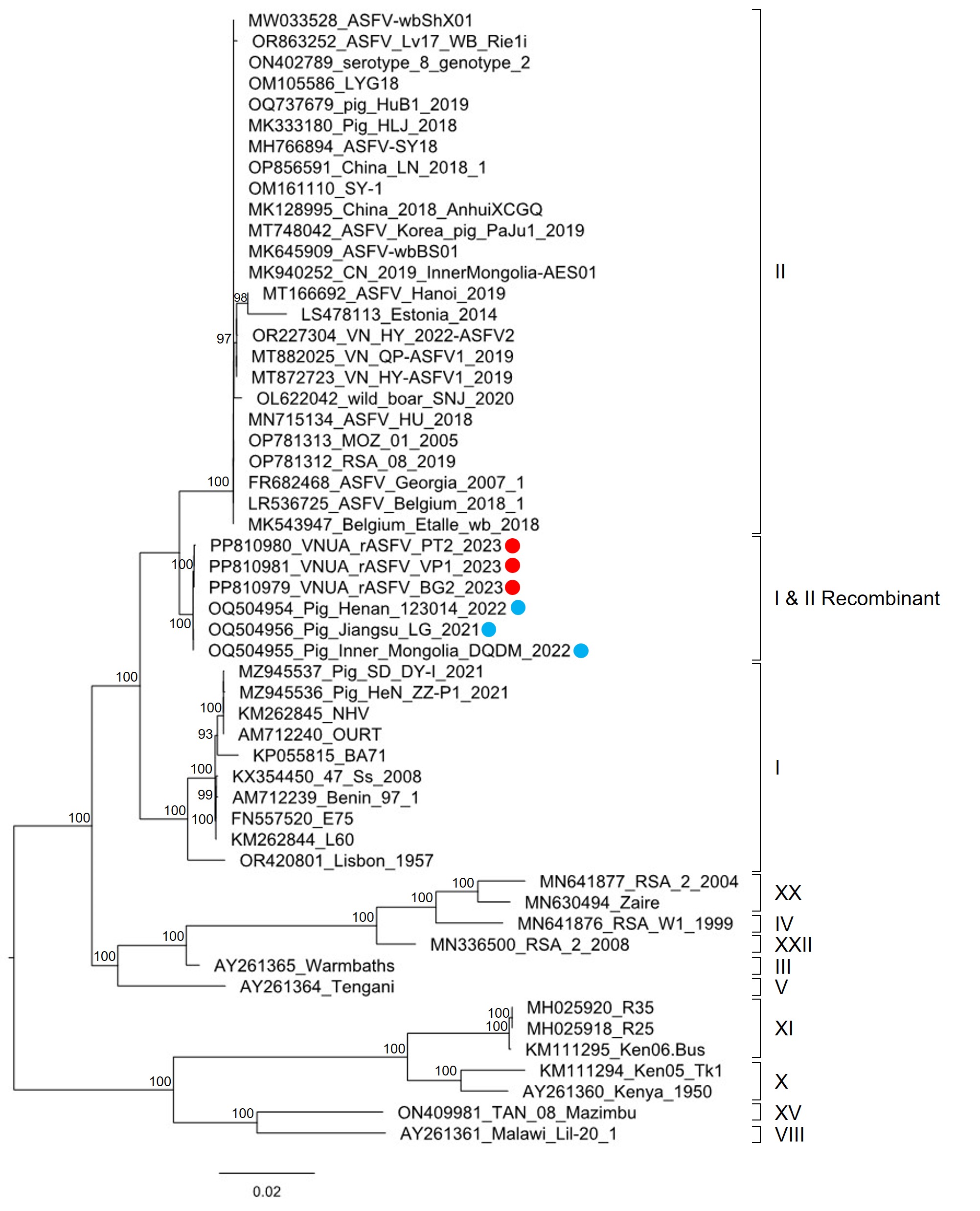

Supplement: Figure S3.jpg [file TEMI_A_2404156_SM7738.jpg]

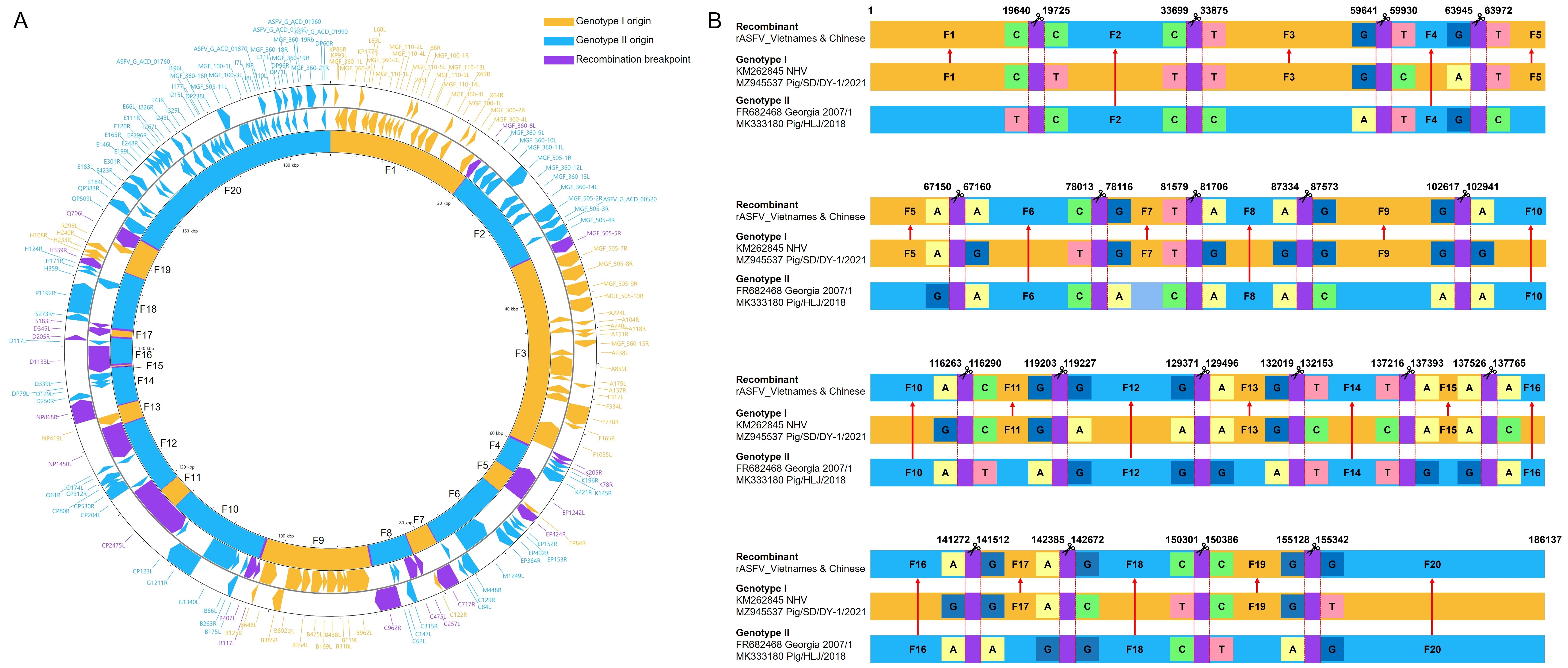

Supplement: Figure S4.jpg [file TEMI_A_2404156_SM7737.jpg]

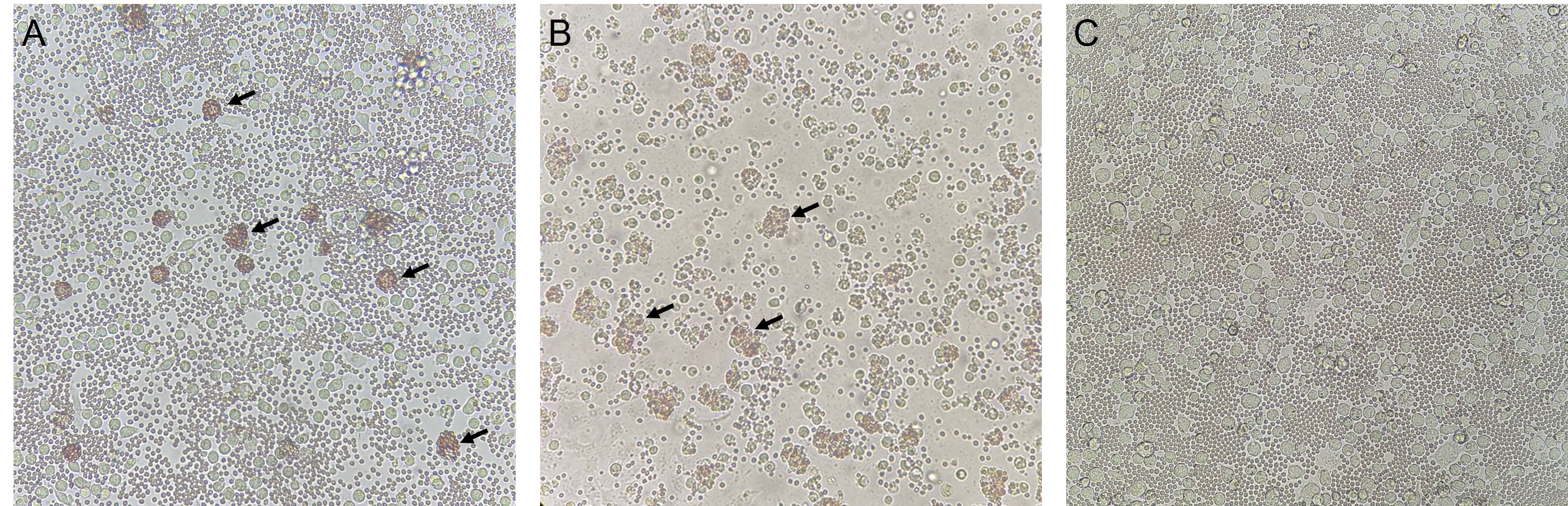

Supplement: Figure S2.jpg [file TEMI_A_2404156_SM7736.jpg]

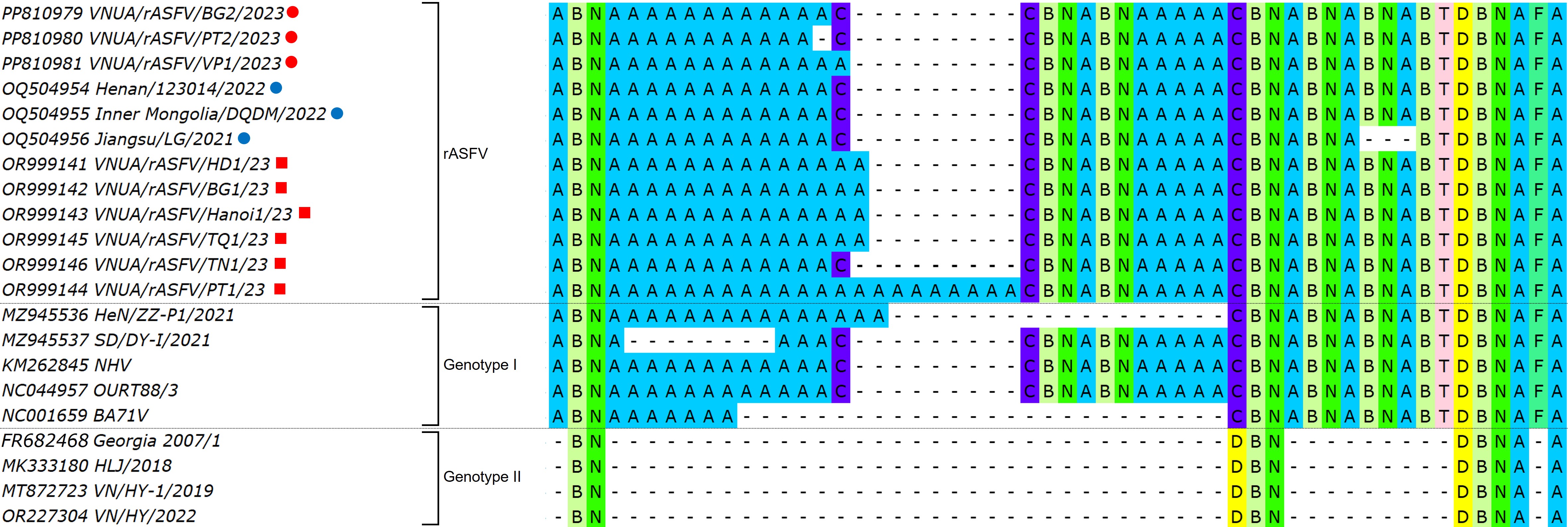

Supplement: Figure S5.jpg [file TEMI_A_2404156_SM7735.jpg]

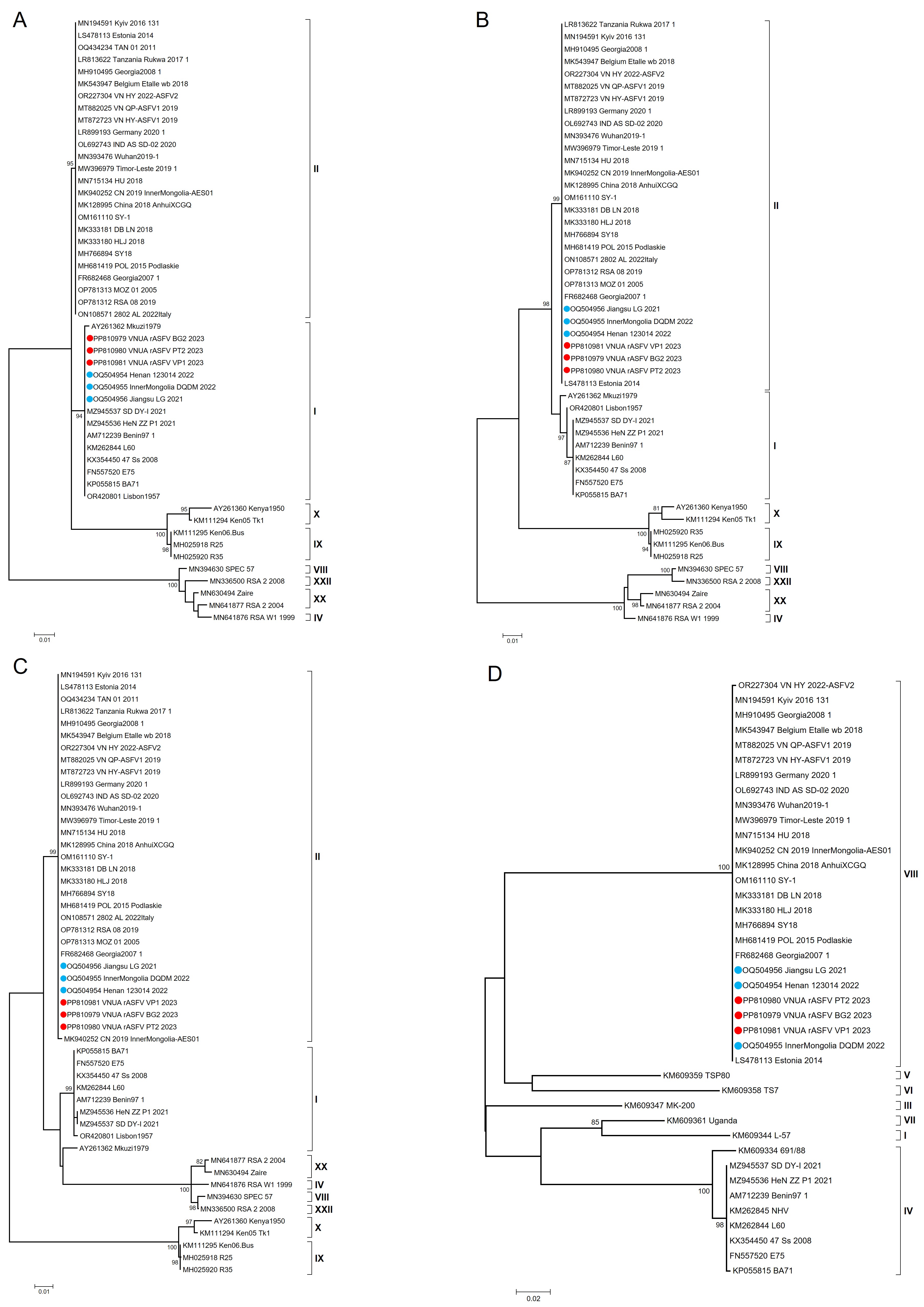

Supplement: Figure S1.jpg [file TEMI_A_2404156_SM7734.jpg]
