# Supplementary material for: Molecular characterization of emerging recombinant African swine fever virus of genotype I and II in Vietnam, 2023
Source: Emerg Microbes Infect. 2024 Sep 11;13(1):2404156. doi: 10.1080/22221751.2024.2404156 (PMC11421136; doi:10.1080/22221751.2024.2404156)
Supplement: Supplementary materials_revision.docx [file TEMI_A_2404156_SM7739.docx]

**Supplementary materials**

**
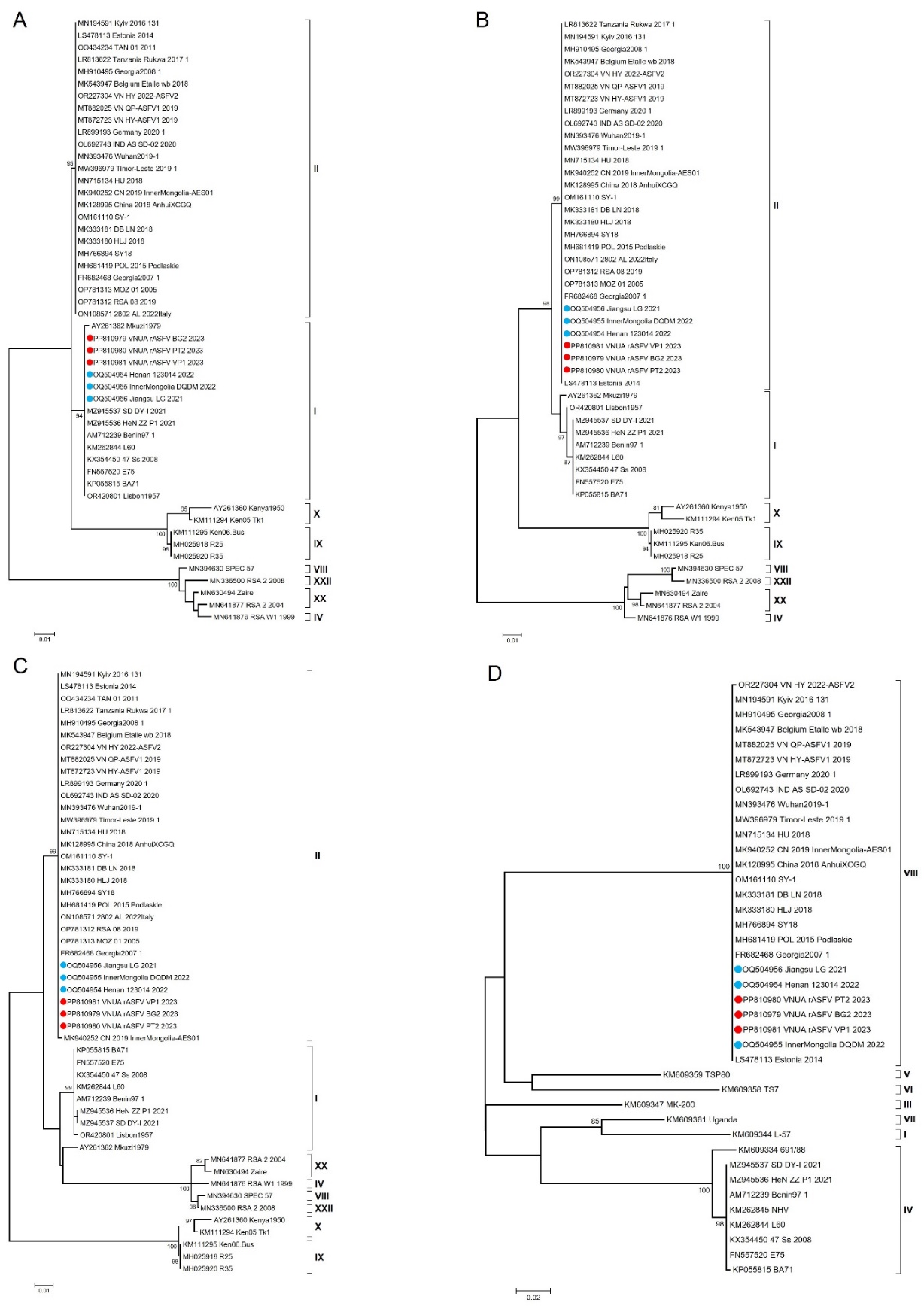
**

**Supplementary Figure S1**. Phylogenetic trees of the African swine fever virus (ASFV) strains based on the (A) B646L, (B) E183L, (C) CP204L, and (D) EP402R genes. Vietnamese (red dots) and Chinese (blue dots) recombinant ASFV strains clustered with genotype I for B646L, genotype II for E183L and CP204L, and serogroup VIII for EP402R gene. Phylogeny trees were constructed by maximum likelihood with 1,000 bootstrap replicates. Nodes show bootstraps >80%. Branch lengths indicate substitutions per site.

**
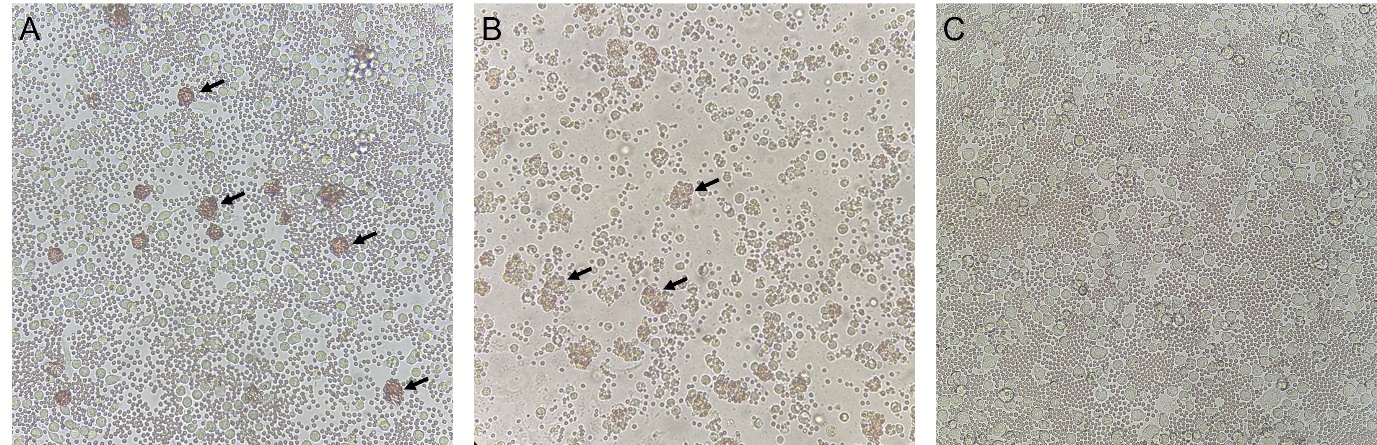
**

**Supplementary Figure S2**. Hemadsorption (HAD) assay results of porcine alveolar macrophages (PAMs) infected with African swine fever virus (ASFV) isolates. (A) PAMs infected with genotype II ASFV strain, showing characteristic rosette formation (arrows) indicative of positive HAD. (B) PAMs infected with recombinant ASFV isolates also exhibiting positive HAD reaction despite being classified as genotype I based on the B646L gene. (C) Uninfected PAMs serving as a negative control.

**
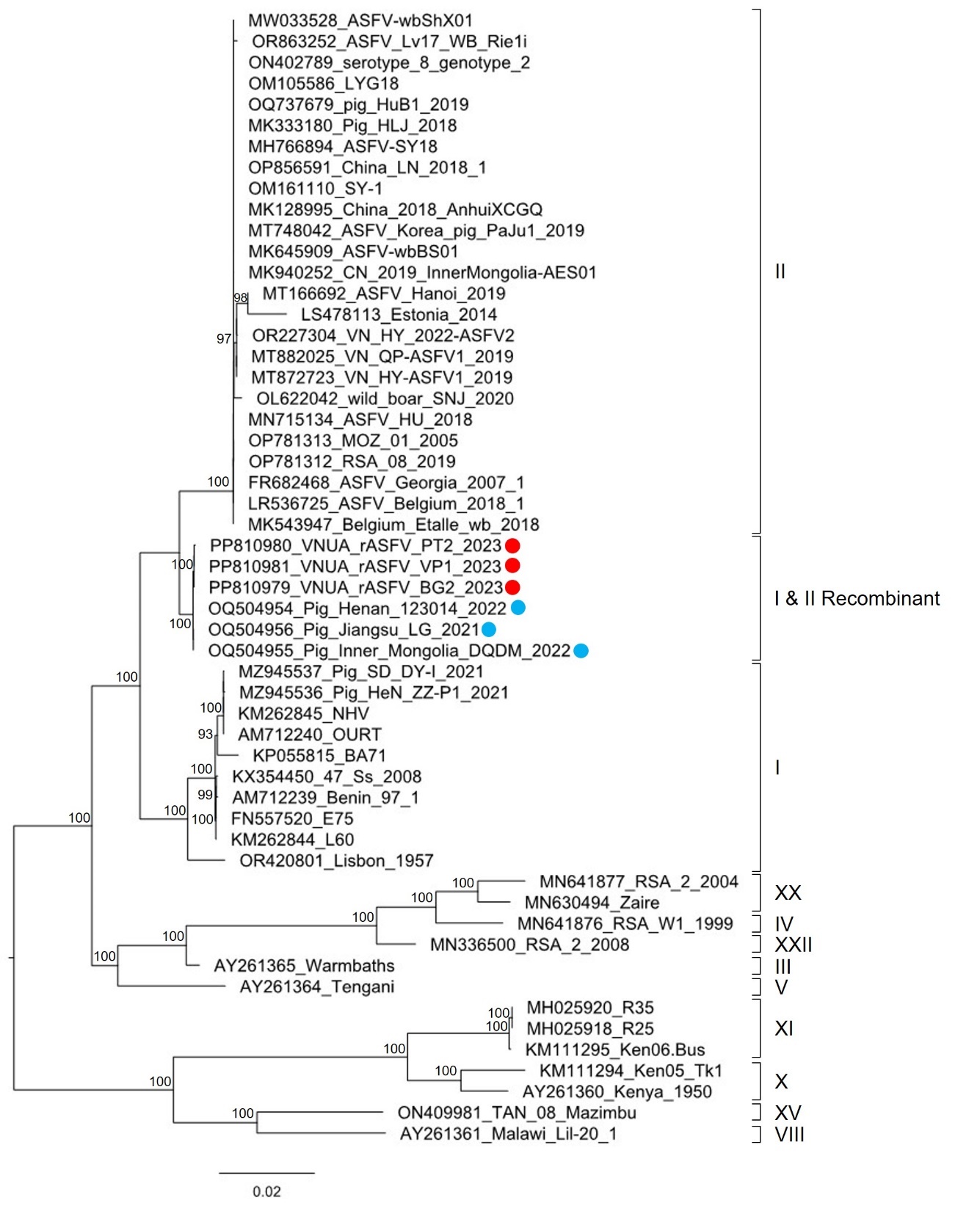
**

**Supplementary Figure S3**. Phylogenetic tree using the whole-genome of African swine fever virus (ASFV). Recombinant ASFV isolates (Vietnamese: red dots, Chinese: blue dots) formed a distinct clade between genotype I and II strains. Phylogeny trees were constructed by maximum likelihood using the GTR+R8+FO model with 1,000 bootstrap replicates. Nodes show bootstraps > 80%. Branch lengths indicate substitutions per site.

**
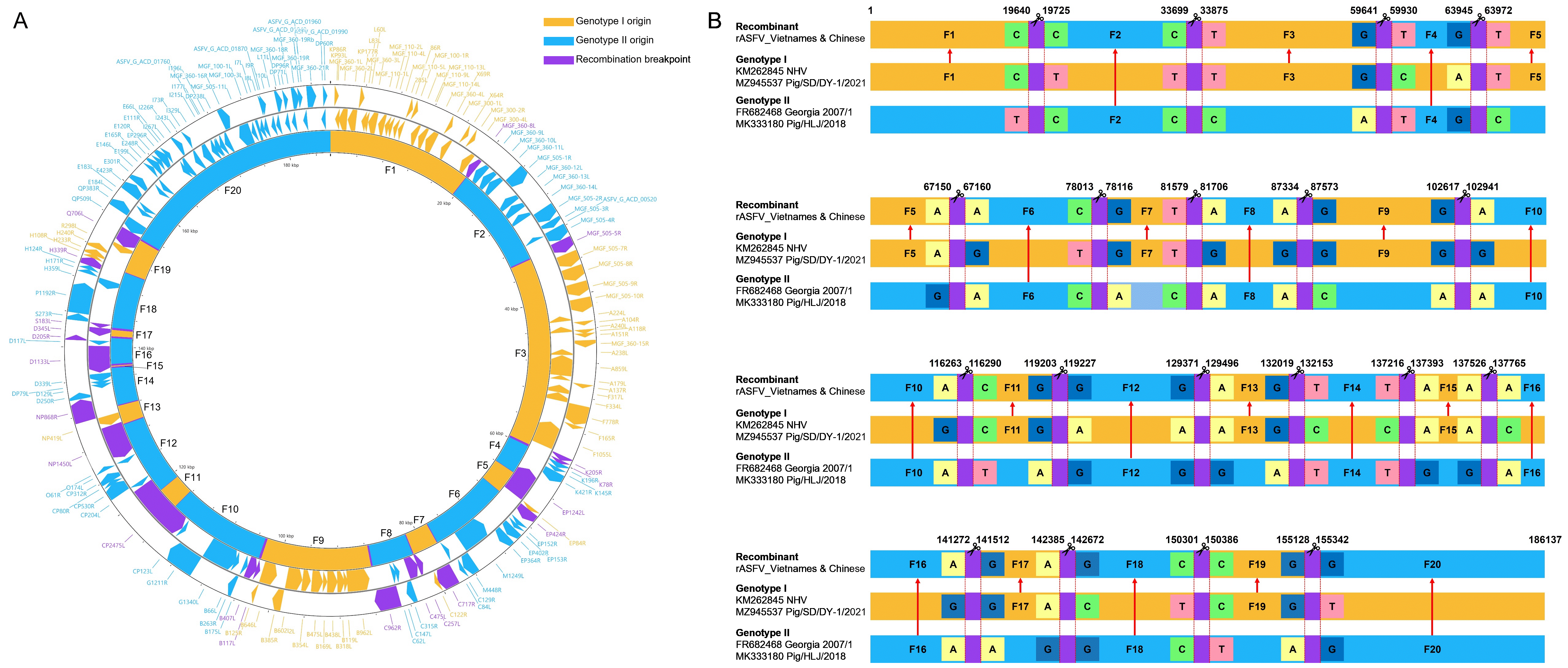
**

**Supplementary Figure S4**. Schematic diagram of the genetic composition of the recombinant African swine fever virus (ASFV) genome. Throughout the figure, the recombinant strain VNUA/rASFV/BG2/2023 is represented, with orange, blue, and purple indicating genotype I origin, genotype II origin, and recombination breakpoints or associated genes, respectively. (A) Circular plots generated using CGView show the open reading frame of the forward (outer circles) and reverse strands (middle circles) and the mosaic structure of the recombinant ASFV genome (inner circles). (B) The linear genome of the recombinant ASFV shows alternating sequence identity with genotypes I and II near the recombination breakpoints.

**
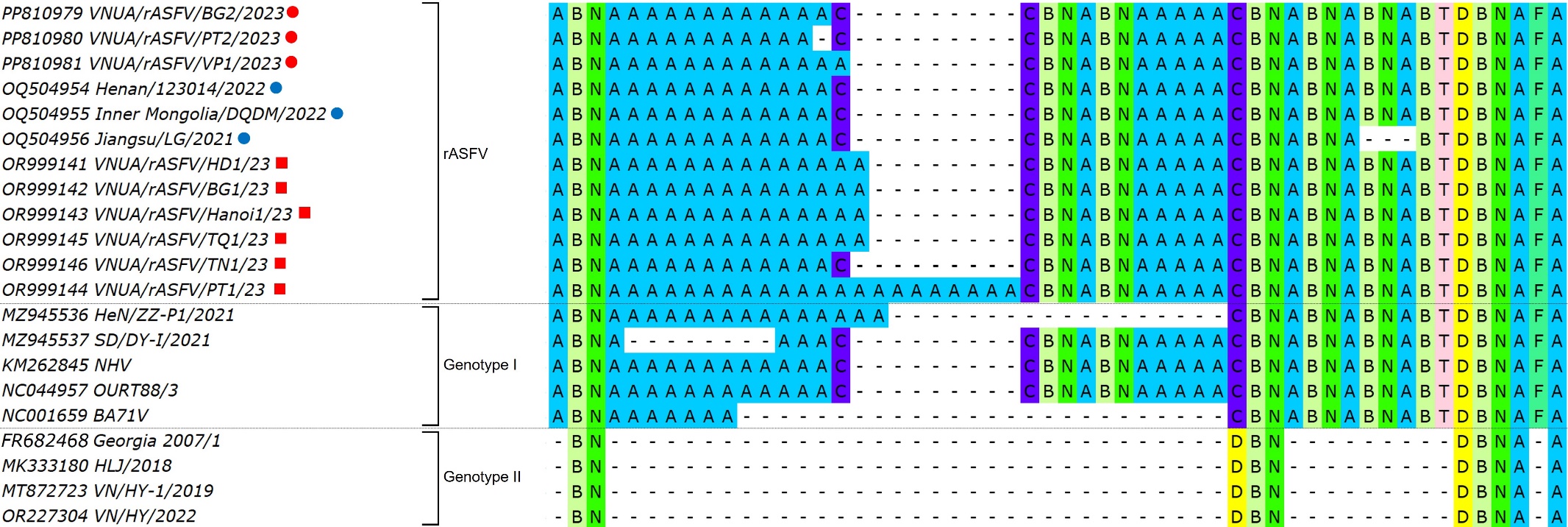
**

**Supplementary Figure S5.** Amino acid sequence alignment of the tetrameric repeats comprising the central variable region of the B602L gene of recombinant African swine fever virus (ASFV). The single letter codes for each tetrameric repeat are: A = CAST/CTST, B = CADT, C = GAST, D = CASM, F = CANT, N = NVDT, T = NVNT. The Vietnamese recombinant ASFV strains in this study (red dots), and previously reported Chinese (blue dots) and Vietnamese (red squares) strains are shown.

**
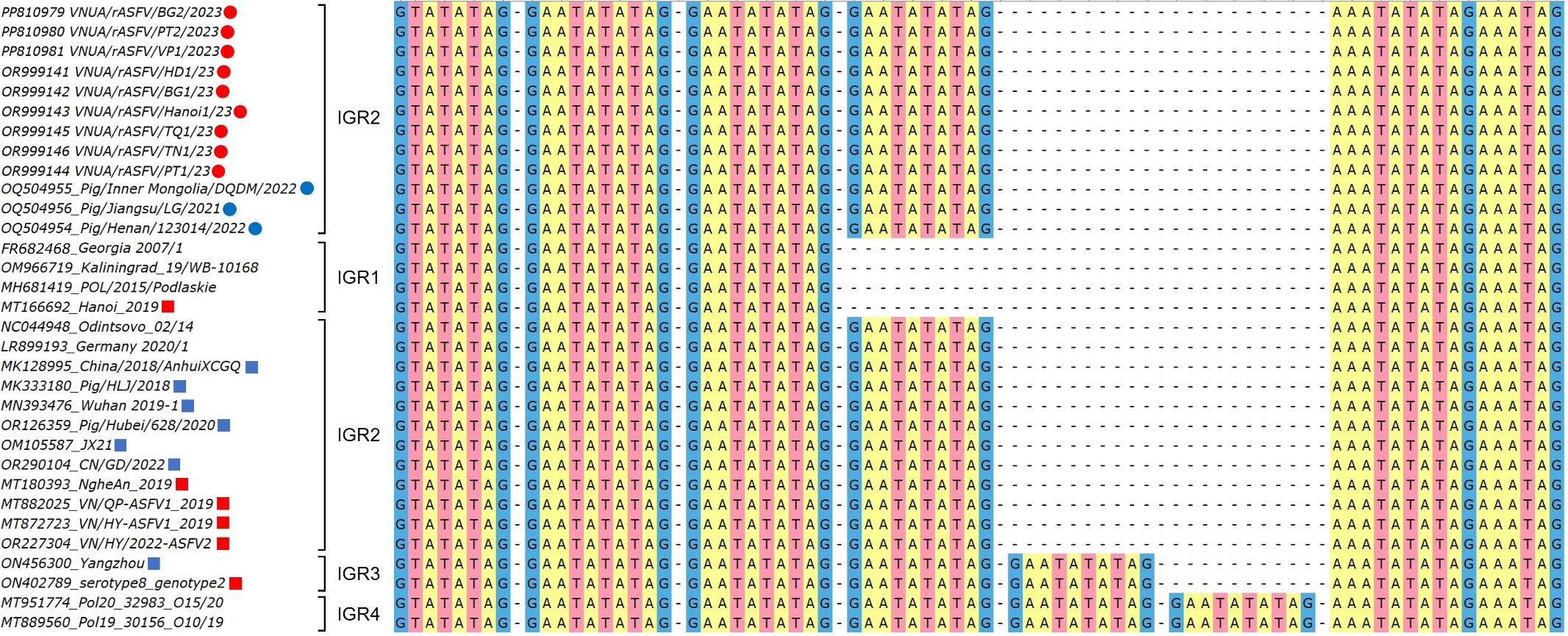
**

**Supplementary Figure S6.** Sequence alignment of the intergenic region between I73R and I329L (I73R/I329L IGR) genes of African swine fever virus (ASFV) strains. The alignment includes sequences from the three Vietnamese recombinant ASFV isolates obtained in this study (VNUA/rASFV/BG2/2023, VNUA/rASFV/PT2/2023, and VNUA/rASFV/VP1/2023; red dots), previously reported Chinese recombinant ASFV strains (blue dots), representative Vietnamese genotype II ASFV strains (red squares), and Chinese genotype II ASFV strains (blue squares). The I73R/I329L IGR sequences of the recombinant ASFV isolates from Vietnam belong to the IGR2 group, consistent with most genotype II ASFV strains circulating in Vietnam and China.

**Supplementary Table S1**. ASFV-positive samples from northern Vietnam, September 2023

| **No.** | **Province** | **Date of collection (dd/mm/yyyy)** | **Specimen Type** | **Ct value** |
| --- | --- | --- | --- | --- |
| 1 | Yen Bai | 06/09/2023 | Whole blood | 15.65 |
| 2 | Bac Giang | 15/09/2023 | Whole blood | 15.97 |
| 3 | Thai Nguyen | 19/09/2023 | Whole blood | 14.58 |
| 4 | Phu Tho | 20/09/2023 | Whole blood | 19.47 |
| 5 | Vinh Phuc | 20/09/2023 | Whole blood | 15.18 |
| 6 | Hai Duong | 23/09/2023 | Whole blood | 17.94 |
| 7 | Hai Duong | 23/09/2023 | Whole blood | 20.02 |

**Supplementary Table S2**. Sequence identity between recombinant ASFV isolates from Vietnam and China

| **Isolates** | **Vietnamese** | | | **Chinese** | | |
| --- | --- | --- | --- | --- | --- | --- |
| **VNUA/rASFV/BG2/2023**  **(PP810979)** | 100 |  |  |  |  |  |
| **VNUA/rASFV/PT2/2023**  **(PP810980)** | 99.833 | 100 |  |  |  |  |
| **VNUA/rASFV/VP1/2023**  **(PP810981)** | 99.944 | 99.876 | 100 |  |  |  |
| **Henan/123014/2022**  **(OQ504954)** | 99.940 | 99.865 | 99.979 | 100 |  |  |
| **Inner Mongolia/DQDM/2022**  **(OQ504955)** | 99.946 | 99.874 | 99.985 | 99.987 | 100 |  |
| **Jiangsu/LG/2021**  **(OQ504956)** | 99.963 | 99.855 | 99.965 | 99.966 | 99.976 | 100 |

*The arrangement of the strains in the rows and columns is identical.

**Supplementary Table S3**. Recombination breakpoints identified in recombinant African swine fever virus (ASFV) strains using Recombination Detection Programme 4 (RDP4) analysis

| **Nucleotide position of recombination event**  **Reference strain: VNUA/rASFV/BG2/2023**  **(GenBank No.PP810979)** | |  | **Average P-value of each detection methods** | | | | | | |
| --- | --- | --- | --- | --- | --- | --- | --- | --- | --- |
| **Begin** | **End** |  | **RDP*** | **GENECONV** | **BootScan** | **MaxChi** | **Chimaera** | **Siscan** | **3Seq** |
| 1 | 19685 |  | 1.708 × 10^−318^ | 9.203 × 10^−304^ | 4504 × 10^−312^ | 1.173 × 10^−63^ | 2.726 × 10^−64^ | 1.330 × 10^−77^ | 1.139 × 10^−12^ |
| 33779 | 59783 |  | 1.597 × 10^−319^ | 2.031 × 10^−303^ | 7.777 × 10^−133^ | 5.462 × 10^−61^ | 2.739 × 10^−61^ | 5.362 × 10^−85^ | 1.139 × 10^−12^ |
| 63957 | 67154 |  | 2.018 × 10^−83^ | 4.568 × 10^−81^ | 2.657 × 10^−28^ | 4.962 × 10−^17^ | 8.460 × 10^−17^ | - | 1.139 × 10^−12^ |
| 78076 | 81675 |  | 4.066 × 10^−49^ | 1.030 × 10^−45^ | 6.151 × 10^−22^ | 2.700 × 10^−9^ | 6.004 × 10^−9^ | - | 1.139 × 10^−12^ |
| 87466 | 102766 |  | 6.581 × 10^−130^ | 7.396 × 10^−127^ | 4.564 × 10^−52^ | 1.643 × 10^−29^ | 2.327 × 10^−29^ | 2.365 × 10^−18^ | 1.139 × 10^−12^ |
| 116276 | 119213 |  | 1.744 × 10^−152^ | 9.145 × 10^−151^ | 2.215 × 10^−150^ | 8.351 × 10^−29^ | 8.701 × 10^−29^ | - | 1.139 × 10^−12^ |
| 129430 | 132091 |  | 2.732 × 10^−45^ | 8.188 × 10^−44^ | 8.518 × 10^−32^ | 3.801 × 10^−7^ | 4.650 × 10^−7^ | - | 7.593 × 10^−13^ |
| 150333 | 155227 |  | 1.125 × 10^−59^ | 6.271 × 10^−58^ | 1.113 × 10^−59^ | 2.373 × 10^−11^ | 2.684 × 10^−11^ | - | 1.139 × 10^−12^ |
| *RDP, Recombination Detection Program | | | | | | | | | |

**Supplementary Table S4**. Mosaic pattern of sequence identity in Vietnamese recombinant African swine fever virus (ASFV) strains compared with genotype I and genotype II ASFV

| **Fragments**  **Reference strain: VNUA/rASFV/BG2/2023**  **(GenBank No. PP810979)** | | |  | **Sequence identity with each genotype (%)** | |
| --- | --- | --- | --- | --- | --- |
| **Number** | **Genomic region** | **Length** |  | **Genotype I SD/DY-1/2021**  **(GenBank No.MZ945537)** | **Genotype II**  **HLJ/2018**  **(GenBank No.MK333180)** |
| 1 | 1–19640 | 19640 |  | **99.94** | 68.94 |
| Recomb. | 19641–19724 | 85 |  | 100 | 100 |
| 2 | 19725–33699 | 13975 |  | 26.33 | **99.99** |
| Recomb. | 33700–33874 | 175 |  | 100 | 100 |
| 3 | 33875–59641 | 25767 |  | **99.99–100** | 95.97 |
| Recomb. | 59642–59929 | 288 |  | 99.65 | 100 |
| 4 | 59930–63945 | 4016 |  | 97.86 | **100** |
| Recomb. | 63946–63971 | 26 |  | 100 | 100 |
| 5 | 63972–67150 | 3179 |  | **100** | 97.55 |
| Recomb. | 67151–67159 | 9 |  | 100 | 100 |
| 6 | 67160–78013 | 10854 |  | 91.72 | **100** |
| Recomb. | 78014–78115 | 102 |  | 100 | 100 |
| 7 | 78116–81579 | 3464 |  | **99.97** | 98.39 |
| Recomb. | 81580–81705 | 126 |  | 100 | 100 |
| 8 | 81706–87334 | 5629 |  | 98.21 | **99.98** |
| Recomb. | 87335–87572 | 238 |  | 100 | 100 |
| 9 | 87573–102617 | 15045 |  | **99.99** | 95.95 |
| Recomb. | 102618–102940 | 323 |  | 100 | 100 |
| 10 | 102941–116263 | 13323 |  | 96.29 | **100** |
| Recomb. | 116264–116289 | 26 |  | 100 | 100 |
| 11 | 116290–119203 | 2914 |  | **100** | 94.48 |
| Recomb. | 119204–119226 | 23 |  | 100 | 100 |
| 12 | 119227–129371 | 10145 |  | 97.78 | **99.99** |
| Recomb. | 129372–129495 | 124 |  | 100 | 100 |
| 13 | 129496–132019 | 2524 |  | **100** | 98.85 |
| Recomb. | 132020–132152 | 133 |  | 100 | 100 |
| 14 | 132153–137216 | 5064 |  | 99.13 | **100** |
| Recomb. | 137217–137392 | 176 |  | 100 | 100 |
| 15 | 137393–137526 | 134 |  | **100** | 97.76 |
| Recomb. | 137527–137764 | 238 |  | 100 | 100 |
| 16 | 137765–141272 | 3508 |  | 98.97 | **100** |
| Recomb. | 141273–141511 | 239 |  | 100 | 100 |
| 17 | 141512–142385 | 874 |  | **99.77** | 99.31 |
| Recomb. | 142386–142671 | 286 |  | 100 | 100 |
| 18 | 142672–150301 | 7630 |  | 98.48 | **100** |
| Recomb. | 150302–150385 | 84 |  | 100 | 100 |
| 19 | 150386–155128 | 4743 |  | **100** | 99.03 |
| Recomb. | 155129–155341 | 213 |  | 100 | 100 |
| 20 | 155342–186137 | 30796 |  | 94.63 | **99.98** |
| Recomb., Recombination breakpoint | | | | | |

**Supplementary Table S5.** Genetic variations identified in the recombinant ASFV strains between Vietnamese and Chinese

| **Variation type**  **(Total Number)** | | | **Nucleotide position and type of mutation** | | | | |
| --- | --- | --- | --- | --- | --- | --- | --- |
|  |  |  | **Reference**  **strain** |  | **Vietnamese strains** | | |
|  |  |  | **IM/DQDM/2022*** |  | **VNUA/BG2/2023** | **VNUA/PT2/2023** | **VNUA/VP1/2023** |
| **SNPs and INDELs**  **(39)** | **IGR**  **(19)** | | 5505 |  | G IN | G DEL | G IN |
|  |  |  | 9245 |  | CCCC IN | CC IN | CCC IN |
|  |  |  | 13567 |  | - | GGG IN | - |
|  |  |  | 13763 |  | GG IN | G DEL | G DEL |
|  |  |  | 15383 |  | GG IN | GG IN | - |
|  |  |  | 15616 |  | GG DEL | G IN | G DEL |
|  |  |  | 15737 |  | G DEL | GG IN | - |
|  |  |  | 15746 |  |  |  | G→T |
|  |  |  | 15747 |  |  |  | T→C |
|  |  |  | 15938 |  | G DEL | GG IN |  |
|  |  |  | 20884 |  | - | G→A | G→A |
|  |  |  | 77230 |  | - | 60nt DEL | - |
|  |  |  | 77251 |  | C→T | - | - |
|  |  |  | 77289 |  | A→G | - | - |
|  |  |  | 77311 |  | C→T | C→T | - |
|  |  |  | 98280 |  | - | G IN | G IN |
|  |  |  | 165459 |  | C→T | - | - |
|  |  |  | 180699 |  | - | G→C | G→C |
|  |  |  | 184120 |  | - | C→T | - |
|  | **CDS**  **(20)** | **Synonymous substitution**  **(9)** | 50700 (F317L) |  | G→A |  |  |
|  |  |  | 64465 (EP1242L) |  | - | A→G | A→G |
|  |  |  | 77667 (C717R) |  | - | G→A | G→A |
|  |  |  | **84799 (C962R)**** |  | **G**→**A** | **G**→**A** | **G**→**A** |
|  |  |  | 87619 (B962L) |  | - | T→G | - |
|  |  |  | 133976 (D250R) |  | C→T | - | - |
|  |  |  | 136641 (D1133L) |  | G→A | - | - |
|  |  |  | 172201 (DP238L) |  | - | G→A | G→A |
|  |  |  | **175048****  **(MGF 505-11L)** |  | **G**→**A** | **G**→**A** | **G**→**A** |
|  |  | **Non-synonymous substitution**  **(11)** | 11596  (MGF 110-14L) |  | - | - | C DEL^†^ (truncated) |
|  |  |  | 35137  (MGF 505-7R) |  | A→G^‡^ (T194A) | A→G^‡^ (T194A) | A→G^‡^ (T194A) |
|  |  |  | 58213 (F1055L) |  | - | - | T→C (D108G) |
|  |  |  | 82261 (C475L) |  | A→T^‡^ (I127N) | A→T^‡^(I127N) | A→T^‡^ (I127N) |
|  |  |  | 94286 (B475L) |  | A→G (S244P) | - | - |
|  |  |  | 97579 (B602L) |  |  |  | C→A(G220C) |
|  |  |  | 97580 (B602L) |  |  |  | T→A(G220C) |
|  |  |  | 97646 (B602L) |  |  | 12nt DEL |  |
|  |  |  | 144099 (P1192R) |  |  | G→A (V200I) |  |
|  |  |  | 150684 (H233R) |  | - | - | A→G (S64G) |
|  |  |  | **168654 (I329L)**** |  | **A**→**T (T839A)** | **A**→**T (T839A)** | **A**→**T (T839A)** |
| SNP, single nucleotide polymorphism; INDEL, insertion and deletion; IGR, intergenic region; CDS, coding sequence  *Inner Mongolia/DQDM/2022 (OQ504955) was used as reference strain  **Molecular markers distinguishing Vietnamese and Chinese recombinant ASFV isolates  ^†^Henan/123014/2022(OQ504954) and Jiangsu/LG/2021(OQ504956) also have the same variant  ^‡^With the exception of Inner Monglia/DQDM/2022, these variants were shared by all recombinant strains | | | | | | | |

**Supplementary Table S6.** Primers used for the validation of single nucleotide polymorphisms (SNPs) and insertions/deletions (INDELs) identified in the Vietnamese recombinant African swine fever virus (ASFV) isolates.

| **Region of SNPs/INDELs**  **(Reference strain: IM/DQDM/2022)** |  | **Orientation** |  | **Primer sequence (5’-3’)** |
| --- | --- | --- | --- | --- |
| 5505-5506 INDELs |  | Forward |  | AGCCCCCAATTTTCGGCATA |
|  |  | Reverse |  | GTTGGACAACACATCTGCCA |
| 9245-9246 INDELs |  | Forward |  | CTACATTCGCCATTCCAGCG |
|  |  | Reverse |  | CCACCGAATCCTTTACGACCA |
| 11596 INDELs |  | Forward |  | ACGTGCACCAGTATTCAAGC |
|  |  | Reverse |  | GGGCCACTCAGTCCTCATTC |
| 13567-13568 INDELs |  | Forward |  | AGTACAGACGTTGCCTATTCGG |
|  |  | Reverse |  | GTATTGCCCAATATTCATTTGGAAC |
| 15383-15738 INDELs and SNPs |  | Forward |  | ACGCCGTTGCATTGAGTACA |
|  |  | Reverse |  | TTTTGATCACTTATAATGCACTGCT |
| 15938-15939 INDELs |  | Forward |  | AGCAGTGCATTATAAGTGATCAAAA |
|  |  | Reverse |  | AGCTACGTTTAATAAAACACTCTGT |
| 20884 SNPs |  | Forward |  | CGCGTTAAGATTTGTGCCGT |
|  |  | Reverse |  | TGAAATGATGCGGATGGCCT |
| 35137 SNPs |  | Forward |  | AGCCAAATTCAGGACTGGCA |
|  |  | Reverse |  | TCTGGGCTGATGGAAAACTCC |
| 50700 SNPs |  | Forward |  | TGTGGACGATGCCTTTGTCT |
|  |  | Reverse |  | TTTTGGCAGCGAGGCTATGA |
| 58213 SNPs |  | Forward |  | GTCGTTTCAAAGGGGGTTGC |
|  |  | Reverse |  | TCCCAAGGAGAAGCCGTAGA |
| 64465 SNPs |  | Forward |  | TCTGTTTACAATGGGGCGCT |
|  |  | Reverse |  | AATGACAGGCGATGACAGCA |
| 77230-77667 INDELs and SNPs |  | Forward |  | CATCCACGTAGACCGTAGCG |
|  |  | Reverse |  | CTCCTCGCTATCACGGTTCC |
| 82261 SNPs |  | Forward |  | ACAGGAACAACGGGAATGGG |
|  |  | Reverse |  | TGCAAGGATGGCTGCGATAA |
| 84799 SNPs |  | Forward |  | GAGGGCGTATTGCGTATGGA |
|  |  | Reverse |  | GCCAGCTTGAGACCAGGAAT |
| 87619 SNPs |  | Forward |  | ACACCCATATGCGAGCCAAA |
|  |  | Reverse |  | TGGACGATGTCCAGCATGAA |
| 94286 SNPs |  | Forward |  | CTCGAATGTGGCACGGGATA |
|  |  | Reverse |  | TATGTGGAGCCGGAGACAGA |
| 97579-97657 SNPs and INDELs |  | Forward |  | TCGGTGTATTCTGTGCTTGC |
|  |  | Reverse |  | AGGGTGGCCTTTTGCTCTTT |
| 98280-98281 INDELs |  | Forward |  | CTTGCTTTTCTGCGTCTGGG |
|  |  | Reverse |  | TTGTTTCGCAGCGTTTCTCC |
| 133976 SNPs |  | Forward |  | GGCTTCTCCCGGATAATGGG |
|  |  | Reverse |  | AATGCGGCATCCTAGTGCTT |
| 136641 SNPs |  | Forward |  | AACCAGCGGATTCCATCCTG |
|  |  | Reverse |  | GATGAGCCAATGGAGCAGGT |
| 144099 SNPs |  | Forward |  | ATTCACAGAGACCACTGCCC |
|  |  | Reverse |  | TGGTCGCCGTATGTATCGTG |
| 150684 SNPs |  | Forward |  | ACGGAACAGCAGCATGAAAAC |
|  |  | Reverse |  | TGTATCAAGGTGGCGTGCAT |
| 165459 SNPs |  | Forward |  | GCAGACCAGCACGTTGAATG |
|  |  | Reverse |  | TGGTGGACAACCATCCCTTC |
| 168654 SNPs |  | Forward |  | CCCCGCTTTGGATACGGAAA |
|  |  | Reverse |  | GCGGGAATAAGCCAGGACAT |
| 172201 SNPs |  | Forward |  | TCGCAATCCTCCTCCTCCAT |
|  |  | Reverse |  | TGCAGAGACAACATGGCTCC |
| 175048 SNPs |  | Forward |  | ACGTATCGAGTACCCCTGCT |
|  |  | Reverse |  | CCCGAGAACGCTTCACAACT |
| 180699 SNPs |  | Forward |  | GCCGCCTCCCCATTATTCTT |
|  |  | Reverse |  | ATGCGCTCGCTTTATTTGCT |
| 184120 SNPs |  | Forward |  | TTGGGGGCTGACATCAATCG |
|  |  | Reverse |  | TCCACCTTATTGGCCGAAGG |

**Supplementary methods**

**Sample collection**

In September 2023, whole blood samples were collected from pig farms suspected of having ASF infection in the northern provinces of Vietnam, namely Yen Bai, Bac Giang, Thai Nguyen, Phu Tho, Vinh Phuc, and Hai Duong. The samples were transported to the Veterinary Biotechnology Core Laboratory, College of Veterinary Medicine, Vietnam National University of Agriculture, Hanoi, Vietnam, for ASFV diagnosis and analysis.

**DNA preparation and molecular diagnostics**

Viral DNA was extracted from whole blood samples using the Patho Gene-spin DNA/RNA extraction kit (Intron Biotechnology, Seoul, Korea, Cat. No. 17154). Molecular diagnosis of ASFV viral DNA using VDx(R) ASFV qPCR Ver 2.0 (Median Diagnostics, Seoul, Korea) targeting the B646L gene was performed as previously described [1].

**Genotyping and serogrouping**

Sanger sequencing and phylogenetic analysis were performed to determine the genotypes (B646L, E183L , and CP204L) and serogroup (EP402R) of ASFV as previously described [2-4]. The resulting amplicons were sent to Macrogen (Seoul, Korea) for Sanger sequencing. Geneious Prime v2024.0.5 (Biomatters, Inc. Boston, MA, USA) was used to assemble the genome sequences, and multiple sequence alignments were performed for each gene using FFT-NS-i in MAFFT version 7.511 [5]. Phylogenetic analysis was performed using MEGA X with the maximum likelihood method [6]. The best-fitting alternative model was selected based on the lowest Bayesian information criterion score, and bootstrap values were calculated using 1,000 replicates.

**Hemadsorption assay**

HAD (Hemadsorption) is used to determine the titer of ASFV strains containing CD2v (EP402R), a gene only found in genotype II and recombinant strains [3,7]. A EP402R gene of ASFV causes porcine red blood cells (pRBCs) to adhere to the surface of infected cells, forming visible clumps that indicate the presence of the virus. PAM cells are prepared in a 96-well plate one day in advance at 10^4^ cells/well concentrations. The virus is serially diluted from 10^-1^ to 10^-7^, and 100 µL of the diluted virus is added to each well with the prepared cells. The virus plate is incubated for 1 hour, then maintenance medium is added. After 24-48 hours, pRBCs are added to each well. HAD is observed daily under an inverted microscope.

**Whole-genome sequencing**

DNA libraries were prepared using a TruSeq Nano DNA Library Preparation Kit (Illumina, San Diego, CA, USA) following the manufacturer's instructions. PCR-enriched fragment sizes were verified using an Agilent Technologies 2100 Bioanalyzer with a DNA 1000 chip (Agilent Technologies, Palo Alto, CA, USA). Library quantification was performed using the Illumina qPCR quantification protocol. Sequencing was conducted on a NovaSeq 6000 (Illumina) with 151 bp paired-end reads. FastQC was used to assess the raw data quality [8], and sequences shorter than 50 bp were filtered out. Low-quality bases (Q-score < 25) were trimmed, and adapters were removed using the Geneious Prime BBDuk plugin.

**Phylogenetic analysis**

The whole genomes of various ASFV genotypic strains were collected from the GenBank database and subjected to multiple sequence alignments using MAFFT 7.511 with the sequences generated in this study. The aligned sequences were then subjected to maximum likelihood phylogenetic tree construction using the IQ-TREE software [9]. The IQ-TREE ModelFinder was used to determine the best nucleotide substitution model. Branch support was assessed using 1,000 ultrafast bootstrap replicates.

**Genome assembly and gene annotation**

Raw reads were processed and assembled using Geneious Prime v2024.0.5. The reads were mapped using Geneious Mapper with the Highest Sensitivity/Slow setting. To fill genomic gaps and validate low-quality regions in the assembled genome, PCR was performed using i-Taq™ DNA Polymerase (Intron Biotechnology, Cat. No. 25021) following the manufacturer's protocol, followed by Sanger sequencing of the PCR products. The Genome Annotation Transfer Utility (GATU) software annotated the assembled genomes [10]. The viral Genome Annotation System detected any open reading frames (ORFs) missed by GATU, followed by a BLAST search against the NCBI database to validate the identified ORFs [11].

**Comparative Nucleotide Sequence Analysis**

***Comparison with reported recombinant ASFV strains***

Genomes of previously reported recombinant ASFV strains from China and Vietnam were obtained from GenBank. Using MAFFT 7.511, we aligned these sequences with our newly generated recombinant ASFV genomes. UGENE software was employed to perform sequence analysis, focusing on identifying SNPs and INDELs in our recombinant ASFV genomes [12]. This initial comparison allowed us to position our new strains within the context of known recombinant ASFVs, highlighting unique genetic features. To validate the identified SNPs and INDELs, we performed Sanger sequencing as previously described [13].

***Comparison to genotype I and II strains***

To understand the origins of different genomic regions in our recombinant strains, we first conducted recombination breakpoint analysis to identify ancestral genotypes for each genomic region. Subsequently, we performed sequence analysis against the identified ancestral genotype for each region. MAFFT 7.511 and UGENE were again utilized for sequence alignment and comparative analysis. To validate the identified SNPs, we performed Sanger sequencing as previously described [13]. This step allowed us to trace the genetic lineage of various genomic segments in our recombinant strains.

***Recombination Breakpoints Analysis***

To accurately identify recombination events, we collected whole genomes of genotype I and genotype II ASFV strains from GenBank. Sequences were aligned using MAFFT 7.511. To reduce redundancy, we removed duplicate sequences with >99.99% identity using CD-HIT 4.8.1 [14]. Recombination Detection Programme 4 (RDP4) was employed to infer recombination breakpoints [15]. RDP4 integrates multiple detection methods: RDP, GENECONV, BootScan, MaxChi, Chimaera, SiScan, and 3Seq. We applied default settings for each algorithm and considered recombination events significant if identified by at least four methods with a p-value <0.05 after Bonferroni correction.

To ensure a comprehensive analysis, we conducted nucleotide sequence analysis in parallel with RDP4 analysis. Regions showing notable deviations in base changes and sequence identity were flagged as potential recombination breakpoints. Final recombination breakpoints were established by integrating results from both nucleotide sequence analysis and RDP4. This multi-pronged approach allowed for a robust identification of recombination events, providing a solid foundation for understanding the genetic structure of our recombinant ASFV strains.

**References**

[1] Le VP, Nguyen VT, Le TB, et al. Detection of Recombinant African Swine Fever Virus Strains of p72 Genotypes I and II in Domestic Pigs, Vietnam, 2023. Emerg Infect Dis. 2024 May;30(5):991-994. doi: 10.3201/eid3005.231775.

[2] Michaud V, Randriamparany T, Albina E. Comprehensive phylogenetic reconstructions of African swine fever virus: proposal for a new classification and molecular dating of the virus. PloS one. 2013;8(7):e69662.

[3] Malogolovkin A, Burmakina G, Tulman E, et al. African swine fever virus CD2v and C-type lectin gene loci mediate serological specificity. Journal of General Virology. 2015;96(4):866-873.

[4] Qu H, Ge S, Zhang Y, et al. A systematic review of genotypes and serogroups of African swine fever virus. Virus Genes. 2022;58(2):77-87.

[5] Katoh K, Rozewicki J, Yamada KD. MAFFT online service: multiple sequence alignment, interactive sequence choice and visualization. Briefings in bioinformatics. 2019;20(4):1160-1166.

[6] Kumar S, Stecher G, Li M, et al. MEGA X: molecular evolutionary genetics analysis across computing platforms. Molecular biology and evolution. 2018;35(6):1547-1549.

[7] Rodríguez JM, Yáñez RJ, Almazán F, et al. African swine fever virus encodes a CD2 homolog responsible for the adhesion of erythrocytes to infected cells. Journal of virology. 1993;67(9):5312-5320.

[8] Andrews S. FastQC: a quality control tool for high throughput sequence data. 2010. 2017.

[9] Trifinopoulos J, Nguyen L-T, von Haeseler A, et al. W-IQ-TREE: a fast online phylogenetic tool for maximum likelihood analysis. Nucleic acids research. 2016;44(W1):W232-W235.

[10] Tcherepanov V, Ehlers A, Upton C. Genome Annotation Transfer Utility (GATU): rapid annotation of viral genomes using a closely related reference genome. BMC genomics. 2006;7:1-10.

[11] Zhang K-Y, Gao Y-Z, Du M-Z, et al. Vgas: a viral genome annotation system. Frontiers in microbiology. 2019;10:184.

[12] Okonechnikov K, Golosova O, Fursov M, et al. Unipro UGENE: a unified bioinformatics toolkit. Bioinformatics. 2012;28(8):1166-1167.

[13] Nguyen VT, Cho K-h, Mai NTA, et al. Multiple variants of African swine fever virus circulating in Vietnam. Archives of Virology. 2022;167(4):1137-1140.

[14] Li W, Godzik A. Cd-hit: a fast program for clustering and comparing large sets of protein or nucleotide sequences. Bioinformatics. 2006;22(13):1658-1659.

[15] Martin DP, Murrell B, Golden M, et al. RDP4: Detection and analysis of recombination patterns in virus genomes. Virus evolution. 2015;1(1):vev003.
